# Association of Sickle Cell Trait with Risk and Mortality of COVID-19: Results from the United Kingdom Biobank

**DOI:** 10.4269/ajtmh.20-1657

**Published:** 2021-06-15

**Authors:** W. Kyle Resurreccion, Joseph Hulsizer, Zhuqing Shi, Jun Wei, Chi-Hsiung Wang, Rong Na, S. Lilly Zheng, Clay Struve, Brian T. Helfand, Janardan Khandekar, Liana Billings, Michael S. Caplan, Jianfeng Xu

**Affiliations:** 1 Program for Personalized Cancer Care, NorthShore University HealthSystem, Evanston, Illinois;; 2 Lake Forest Academy, Lake Forest, Illinois;; 3 CSS LLC, Chicago, Illinois;; 4 Department of Surgery, University of Chicago Pritzker School of Medicine, Chicago, Illinois;; 5 Center for Molecular Medicine, NorthShore University HealthSystem, Evanston, Illinois;; 6 Department of Medicine, NorthShore University HealthSystem, Evanston, Illinois; 7 Department of Medicine, University of Chicago Pritzker School of Medicine, Chicago, Illinois;; 8 Department of Pediatrics, NorthShore University HealthSystem, Evanston, Illinois

## Abstract

Sickle cell trait (SCT) carriers inherit one copy of the Glu6Val mutation in the hemoglobin gene and is particularly common in Black individuals (5–10%). Considering the roles of hemoglobin in immune responses and the higher risk for coronavirus disease (COVID-19) among Black individuals, we tested whether Black SCT carriers were at increased risk for COVID-19 infection and mortality according to the United Kingdom Biobank. Among Black individuals who were tested for COVID-19, we found similar infection rates among SCT carriers (14/72; 19.7%) and noncarriers (167/791; 21.1%), but higher COVID-19 mortality rates among SCT carriers (4/14; 28.6%) than among noncarriers (21/167; 12.6%) (odds ratio [OR], 3.04; 95% confidence interval [CI], 0.69–11.82; *P* = 0.12). Notably, SCT carriers with preexisting diabetes had significantly higher COVID-19 mortality (4/4; 100%) than those without diabetes (0/10; 0%; (OR, 90.71; 95% CI, 5.66–infinite; *P* = 0.0005). These findings suggest that Black SCT carriers with preexisting diabetes are at disproportionally higher risk for COVID-19 mortality. Confirmation by larger studies is warranted.

## INTRODUCTION

The coronavirus (COVID-19) pandemic has impacted communities worldwide, but its effects are disproportionately expressed in certain groups. The Center for Disease Control reported that non-Hispanic Black individuals are approximately 1.4-times and 2.8-times more likely to test positive for and die of COVID-19, respectively, compared with White individuals.^1^ This finding is supported by United Kingdom Biobank (UKB) studies, which found significantly higher positive rates among Black individuals even after adjusting for biological, behavioral, and socioeconomic factors.^2,3^ Additionally, evidence shows that individuals with chronic conditions such as cardiovascular disease, diabetes, chronic respiratory disease, hypertension, and sickle cell disease (SCD) are particularly vulnerable to more severe COVID-19 disease.^1,4^

The intersection of race and SCD makes it important to consider their impact on COVID-19. SCD is a genetic disorder whereby patients inherit two pathogenic mutations of the gene that codes for hemoglobin (Hgb). It mostly affects people of sub-Saharan African descent; therefore, it is common in many Black communities worldwide.^5,6^ Although SCD patients are relatively rare, individuals with only one pathogenic mutation, called sickle cell trait (SCT) carriers, are more common. Compared with SCD, which is associated with serious health conditions,^6–9^ most SCT carriers are considered asymptomatic. However, approximately 40% of the Hgb in carriers can induce sickling under certain conditions.^5,6^ Additionally, recent evidence suggests that Hgb may also have a modulatory role in the immune response against RNA viruses, including severe acute respiratory syndrome coronavirus 2 (SARS-CoV-2).^10,11^

Because of the novelty of the COVID-19 pandemic, it is still unknown how SCT carriers are uniquely affected by the virus because of defective Hgb. The larger number of carriers compared with SCD patients makes studying this condition more relevant to a larger population and more feasible (increased statistical power from larger sample sizes). Therefore, the goal of this study was to examine the association of COVID-19 and SCT carriers. Based on the role of Hgb in immune responses and higher risk for COVID-19 among Black individuals, we hypothesized that SCT was associated with an increased risk for COVID-19 and mortality, especially for Black individuals. Furthermore, we hypothesized that other candidate genetic and lifestyle risk factors as well as comorbidities may modify the risk of COVID-19 for SCT carriers.

## METHODS

This study was performed using the UKB, a population-based study with extensive genetic and phenotypic data for approximately 500,000 individuals cross the United Kingdom 40 to 69 years of age at recruitment.^12^ Extensive phenotypic and health-related information was available for each participant, including questionnaire answers, biological measurements, lifestyle indicators, and biomarkers in blood and urine. Follow-up information was provided by linking health and medical records. Candidate comorbidities and risk factors [body mass index (BMI), diabetes, chronic obstructive pulmonary disease, hypertension, cancer, stroke, and smoking status] were obtained through the International Classification of Diseases-10 (ICD-10) codes and self-report.

The UKB Axiom SNP array data were available for all subjects, whereas whole-exome sequencing (WES) data were available for approximately 40% of subjects. For subjects without WES data, SCT carriers were identified based on ICD codes alone (i.e., D57.3). Codes for SCD (D57.0, D57.1, D57.2, and D57.8) and β-thalassemia (D56.1) were excluded. For subjects with WES data, carriers were identified using a combination of ICD codes and heterozygous Glu6Val mutations in the *HBB* gene. Subjects with ICD codes for SCD, homozygous Glu6Val, or double heterozygous (heterozygous Glu6Val mutation plus any known mutations in *HBB* such as Glu121Lys, Glu121Gln, Asp73Asn, and Val23Ile) were considered to have SCD and removed from the remaining analyses.^13^ Blood types (ABO) were inferred from rs8176746 and rs8176719 genotypes,^14^ and APOE alleles were inferred from rs7412 and rs429358 genotypes (ε2 = T/T, ε3 = C/T, and ε4 = C/C).^15^

The COVID-19 test results for the UKB participants were provided by Public Health England (for participants residing in England). This information was updated weekly. Additional COVID-19 data are also collected through general practitioner (primary care) data, hospital inpatient data, death data, and critical care data (for COVID-19-positive patients); these data are updated monthly.

Logistic regression analysis with the maximum likelihood estimation method was used to test the associations of SCT and COVID-19 positivity/mortality and adjusted for other known demographic variables (race, age at testing, BMI, and sex). An exact logistic regression analysis was used when the sample size was small (total sample size < 40); we modeled the log odds of binary outcomes as a linear combination of the predictor variables. The estimates provided by exact logistic regression do not depend on asymptotic results caused by small cells. The model diagnosis and goodness of fit were examined using Hosmer and Lemeshow’s test. Specific models used for each test are described in each table.

## RESULTS AND DISCUSSION

Based on the ICD codes and mutations in the *HBB* gene, 729 subjects recorded in the UKB were classified as SCT carriers. Consistent with previous reports,^5,6^ most of the carriers were Black (571/729; 78%). The estimated carrier rate by race was highest for Black individuals (571/8,017; 7.1%) and considerably lower for other races (Asian, 11/11,399 [0.1%]; White, 20/471,180 [0.004%]; mixed race, 40/2,928 [1.4%]; and other, 69/4,605 [1.5%]). It was noted that the carrier rate was significant higher for females, especially those younger than 45 years at the time of recruitment. For Black individuals, for example, the carrier rate was 7.8% (360/4,633) for females and 6.2% (211/3,384) for males overall (*P* = 0.009); however, it was 8.9% (82/920) for females and 4.6% (36/783) for males among subjects younger than 45 years at the time of recruitment (*P* = 0.0007) (Supplemental Table 1). This novel observation warrants confirmation and may suggest higher mortality for men at younger ages.

By December 21, 2020, COVID-19 test results were available for 44,724 subjects (approximately 8.9% [44,724/500,822] of the UKB population). Among those tested for COVID-19, 17.5% (7,849/44,724) had positive results; of these, 6.3% (495/7,849) died of COVID-19 (Table 1). Black individuals had nonsignificantly higher COVID-19 positivity rates (21.0% [181/862] compared to 17.2% [7,167/41,660]; *P* = 0.94) but significantly higher COVID-19 death rates than White individuals (13.8% [25/181] compared to 6.2% [445/7,167]; *P* < 0.0001). The mean age at the time of the COVID-19 diagnosis was also significantly younger for Black individuals (64.5 years) than for White individuals (69.7 years) (*P* < 0.0001). Similarly, the mean age at the time of death attributable to COVID-19 was significantly younger for Black individuals (71.1 years) than for White individuals (75.1 years) (*P* < 0.0001). Most of these findings are consistent with those of other published studies.^2,3^

**Table 1:** Association of SCT with COVID-19 positivity and mortality in the UK Biobank

	Subjects	No. (%) tested for COVID-19	Positive for COVID-19			Died of COVID-19		
			Subjects	OR (95% CI)*	*P* value*	Subjects	OR (95% CI)*	*P* value*
All	500,822	44,724 (8.9%)	7,849 (17.5%)			495 (6.3%)		
By race								
White individuals	471,180	41,660 (8.8%)	7,167 (17.2%)	Ref*		445 (6.2%)	Ref*	
Black individuals	8,017	862 (10.8%)	181 (21%)	0.99 (0.84–1.18)	0.95	25 (13.8%)	6.3 (3.76–10.25)	5.19E-13
By SCT status								
No SCT	500,093	44,631 (8.9%)	7,828 (17.5%)	Ref†		491 (6.3%)	Ref†	
SCT	729	93 (12.8%)	21 (22.6%)	1.12 (0.66–1.84)	0.67	4 (19%)	2.87 (0.69–9.95)	0.11
Black individuals								
No SCT	7,446	791 (10.6%)	167 (21.1%)	Ref†		21 (12.6%)	Ref^*^	
SCT	571	71 (12.4%)	14 (19.7%)	0.98 (0.51–1.78)	0.96	4 (28.6%)	3.04 (0.69–11.82)	0.12

CI = confidence interval; OR = odds ratio; SCT = sickle cell trait.

* Standard logistic regression analysis adjusted for age at COVID-19 test and sex.

† Standard logistic regression analysis adjusted for age at COVID-19 test, sex, and race.

Compared with individuals who were not SCT carriers, SCT carriers had similar COVID-19 positivity rates but higher COVID-19 mortality rates overall among Black individuals (Table 1). For Black individuals, the positivity rates were 19.7% (14/71) and 21.1% (167/791) for subjects with SCT and without SCT (*P* = 0.96). The death rate was higher, but not statistically significant, for Black SCT carriers (4/14; 28.6%) than for Black noncarriers of SCT (21/167; 12.6%) (odds ratio [OR], 3.04; 95% confidence interval [CI], 0.69–11.82; *P* = 0.12).

We also examined whether other candidate demographic and genetic factors as well as comorbidities affected COVID-19 positivity and mortality of Black SCT carriers (*N* = 71).^1,2,4,15^ Most of these variables were not significantly associated with COVID-19 positivity and mortality (Table 2), partially because of the small sample size. However, comorbid diabetes (type 1 diabetes and type 2 diabetes) was more common in SCT carriers who were positive for COVID-19 (4/14; 28.6%) than in SCT carriers who were negative for COVID-19 (7/57; 12.3%) (OR, 2.81; 95% CI, 0.51–13.80; *P* = 0.21). In particular, diabetes was significantly more common in SCT carriers who died of COVID-19 (4/4; 100%) than in those who survived (0/10; 0%) (OR, 90.71; 95% CI, 5.66–infinite; *P* = 0.0005).

**Table 2:** Candidate variables affecting the association of SCT with COVID-19 positivity and mortality in Black individuals of the UK Biobank

Variables	SCT carriers				SCT carriers with COVID-19			
	COVID-19-positive (*N* = 14)	COVID-19- negative (*N* = 57)	OR (95% CI)	*P* value	Died of COVID-19 (*N* = 4)	Remained alive (*N* = 10)	OR (95% CI)	*P* value
Demographic factors								
Age, mean (SD)	64.37 (8.05)	64.26 (8.66)	1 (0.93–1.07)	0.97	73.86 (4.66)	60.57 (5.5)	1.98 (1.11–11.62)	0.009
Sex, no. of females	8 (57.1%)	46 (80.7%)	0.32 (0.08–1.38)	0.08	4 (100%)	4 (40%)	0.17 (0–1.66)	0.08
Smoking, current/ previous	2 (14.3%)	18 (31.6%)	0.36 (0.04–1.87)	0.32	1 (25%)	1 (10%)	2.68 (0.03–238.74)	0.50
BMI, mean (SD)	30.09 (4.85)	29.99 (4.8)	1 (0.88–1.14)	0.94	32.4 (3.34)	29.06 (5.22)	1.15 (0.89–1.56)	0.33
Genetic factors								
APOE, ε4-positive, n	3 (21.4%)	19 (33.3%)	0.62 (0.10–2.88)	0.74	2 (50%)	1 (10%)	9.84 (0.35–770.19)	0.12
ABO, type O, n	7 (50%)	28 (49.1%)	1.04 (0.31–3.52)	> 0.99	1 (25%)	6 (60%)	0.37 (0.00–9.28)	0.57
Comorbidity								
Diabetes, n	4 (28.6%)	7 (12.3%)	2.81 (0.51–13.80)	0.21	4 (100%)	0 (0%)	90.71 (5.66–inf)	4.70E-04
Obesity (BMI ≥ 30), n	6 (42.9%)	28 (49.1%)	0.86 (0.26–2.87)	> 0.99	3 (75%)	3 (30%)	4.94 (0.29–312.26)	0.26
Chronic obstructive pulmonary disease, n	2 (14.3%)	9 (15.8%)	0.89 (0.08–5.19)	> 0.99	0 (0%)	2 (20%)	1.04 (0–14.58)	> 0.99
Hypertension, n	10 (71.4%)	32 (56.1%)	1.94 (0.48–9.48)	0.37	4 (100%)	6 (60%)	2.95 (0.28–inf)	0.24
Cancer diagnosis, n	0 (0%)	8 (14%)	0 (0.00–2.36)	0.34	0 (0%)	0 (0%)		
Stroke, n	0 (0%)	1 (1.8%)	0 (0.00–158.40)	> 0.99	0 (0%)	0 (0%)		

APOE = apolipoprotein E; BMI = body mass index; CI = confidence interval; inf = infinite; OR = odds ratio; SCT = sickle cell trait; SD = standard deviation. The analysis of variance test was used for continuous variables. Fisher’s exact test was used when the expected number of subjects in any cell was < 5.

The effect of diabetes comorbidity on COVID-19 has been reported previously.^1,4^ During the current study, we found diabetes was significantly associated with a higher risk of death cause by COVID-19 (OR, 1.75; 95% CI. 1.32–2.31; *P* < 0.0001) (Table 3). The risks for COVID-19 positivity and mortality were higher for Black diabetes patients, and especially for Black SCT carriers. For example, all four Black SCT carriers died of COVID-19, but 6 of 34 (17.6%) Black noncarriers of SCT died of COVID-19 (OR, 9.02; 95% CI, 0.74–infinite; *P* = 0.05).

**Table 3:** Sequential stratified association tests for COVID-19 positivity and mortality with diabetes, race, and SCT in the UK Biobank

	Subjects	Subjects (%) tested for COVID-19	Positive for COVID-19			Died of COVID-19		
			Subjects (%)	OR (95% CI)	*P* value	Subjects (%)	OR (95% CI)	*P* value
By diabetes status								
No diabetes	469,225	40,371 (8.6%)	7,114 (17.6%)	Ref*		379 (5.3%)	Ref*	
Diabetes	31,597	4,353 (13.8%)	735 (16.9%)	0.98 (0.9–1.07)	0.69	116 (15.8%)	1.75 (1.32–2.31)	7.78E-05
By race with diabetes								
White individuals	27,683	3,771 (13.6%)	599 (15.9%)	Ref†		95 (15.9%)	Ref†	
Black individuals	978	156 (16.0%)	38 (24.4%)	1.71 (1.15–2.49)	6.10E-03	10 (26.3%)	3.92 (1.62–9.07)	1.67E-03
By SCT with diabetes for Black individuals								
No SCT	895	145 (16.2%)	34 (23.4%)	Ref†		6 (17.6%)	Ref‡	
SCT	83	11 (13.3%)	4 (36.4%)	3.2 (0.74–12.74)	0.1	4 (100%)	9.02 (0.74–inf)	0.05

APOE = apolipoprotein E; BMI = body mass index; CI = confidence interval; inf = infinite; OR = odds ratio; SCT = sickle cell trait; SD = standard deviation.

* Standard logistic regression analysis adjusted for age at COVID-19 test, sex, BMI, and race.

† Standard logistic regression analysis adjusted for age at COVID-19 test, BMI, and sex.

‡ Exact logistic regression analysis adjusted for age at COVID-19 test and sex.

A unique strength of this study was our better ability to identify SCT carriers in the population using genetic information. We found that SCT is considerably under-reported by ICD codes. Among subjects with WES data in our study, we found the majority of SCT carriers defined by heterozygous Glu6Val did not have an ICD code for SCT (409/527; 77.6%). This finding has two important implications. First, many SCT carriers may be misclassified if they are without WES data, which would create a bias toward a null finding. Second, and probably more important, our data suggest that the SCT carrier rate is considerably higher than that previously estimated, and the majority of SCT carriers in the general population are not aware of their status. Therefore, many SCT carriers miss the opportunity for extra care during the prevention and management of COVID-19.

One limitation of this study was the relatively small sample size. However, this study represents one of the largest available studies of the association between COVID-19 and SCD/SCT.^7–9^ Confirmation of our findings by additional study populations is needed. Another limitation was the lack of detailed clinical information regarding COVID-19 symptoms and treatment. This limited our ability to perform a more comprehensive analysis. Nevertheless, because of the novel and emergent status of this pandemic, more data will likely become available for additional analyses by future studies to address both limitations of this study.

In conclusion, in this first study testing the association of SCT and COVID-19 using a population-based cohort, we found that Black SCT carriers had a trend of higher, albeit not statistically significant, COVID-19 mortality than noncarriers of SCT. We also found that Black SCT carriers with pre-existing diabetes had significantly higher COVID-19 mortality than those without diabetes. Considering that the majority of SCT carriers may not be aware of their condition, and considering that Black individuals are already at disproportionally higher risk for both COVID-19 and diabetes,^16^ these findings suggest special care during the prevention and management of COVID-19 should be considered for Black SCT carriers.
